# Extra-Anatomical Bypass: A Surgical Option for Recurrent Aortic Coarctation

**DOI:** 10.1155/2013/320132

**Published:** 2013-07-10

**Authors:** Alban Malaj, Ombretta Martinelli, Francesco Giosue' Irace, Jihad Jabbour, Bruno Gossetti, Giuseppe Mazzesi

**Affiliations:** ^1^Division of Vascular Surgery, Paride Stefanini Department, Policlinico Umberto I Hospital Sapienza, University of Rome, Viale del Policlinico 155, 00161 Rome, Italy; ^2^Division of Cardiac Surgery, Paride Stefanini Department, Policlinico Umberto I Hospital Sapienza, University of Rome, Viale del Policlinico 155, 00161 Rome, Italy

## Abstract

*Background*. Balloon aortoplasty with or without stenting is a less invasive alternative to open surgery for the management of recurrent isthmic coarctation. However, in patients with previous small size tube graft, an open surgical correction is mandatory and, in most cases, an anatomical aortic reconstruction is carried out. *Methods*. We present the case of a 48-year-old woman with recurrent aortic coarctation and systemic hypertension with systolic value around 190–200 mmHg and preoperative systolic pressure gradient 70 mmHg, submitted to an extra-anatomical bypass. Through a median sternotomy, an extra-anatomical bypass from ascending to descending aorta was performed. *Results*. No intra- or postoperative complications were observed. The postoperative pressure gradient was 10 mmHg and the systolic pressure ranged from 130 to 140 mmHg. *Conclusion*. The extra-anatomical bypass can be considered an effective and safe alternative to the anatomical aortic reconstruction in the cases with recurrent aortic coarctation unfit for endovascular treatment.

## 1. Introduction

The incidence of isthmic coarctation of aorta is 6-7% of congenital heart disease [1]. Surgical treatment is performed in order to avoid serious heart complications. In fact not-operated patients early develop severe hypertension and heart failure. Surgical options in patients without other abnormalities include patch aortoplasty, subclavian flap angioplasty, combination of end-to-end anastomosis, tube graft. These interventions are carried out mostly in pediatric age and the incidence of recurrent coarctation ranges between 5 and 50% in relation to the diagnostic criteria [2]. It is the age of the first intervention that affects the recurrence frequency and less important are the various types of operations used.

Undoubtedly the use of small-caliber prostheses in children or young patients increases the need for reoperation. In recent years, the balloon aortoplasty with or without stenting is a less invasive alternative to open surgery but can determine many complications and now this technique needs further experience to be validated [3]. In patients with recurrent coarctation the surgical treatment is also related to the onset of hypertension, ventricular hypertrophy, and dilation. The choice of the operation is dictated by the patient's general conditions as well as by age and type of the previous surgical treatment. Balloon aortoplasty is not feasible in patients with previous tube graft interposition at their first operation. 

Open aortic reconstruction is often very difficult due to fibrous involvement of perigraft and periaortic tissue. We report a case of recurrent coarctation in a woman treated by an extra-anatomical bypass from ascending to descending aorta.

## 2. Case Report

**Figure 1:**
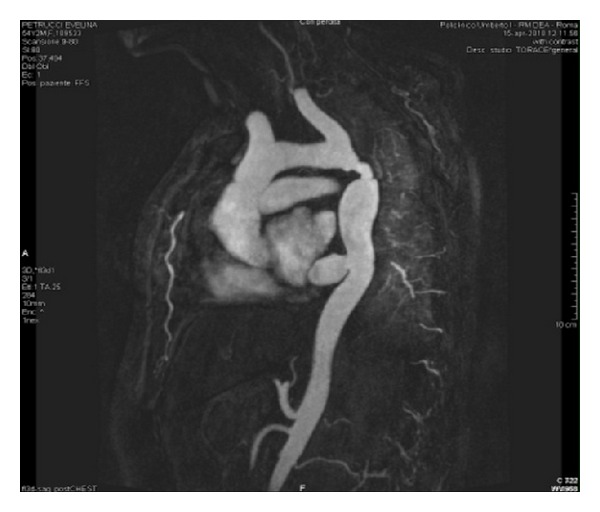
MR, recurrent aortic coarctation.

**Figure 2:**
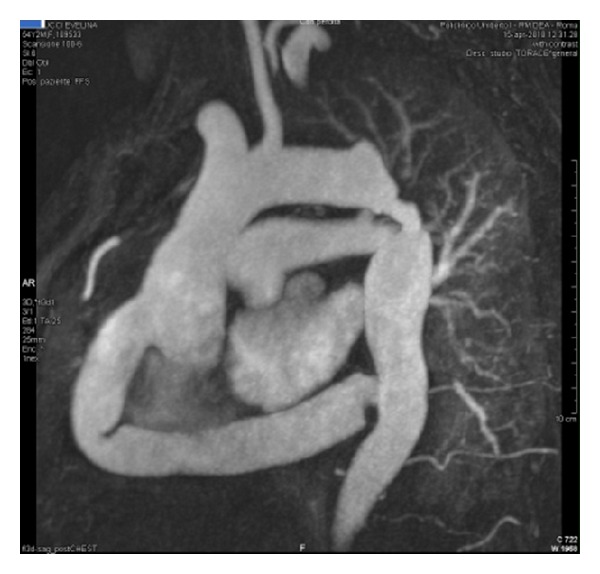
Six-month MR: extra-anatomical bypass from ascending to descending aorta.

A 48-year-old woman was admitted to our division for recurrent aortic coarctation. At the age of sixteen, she underwent previous operation. A Dacron tube graft, 12 mm in size, was employed to correct a primitive isthmic aortic coarctation. The patient was in good conditions for about 25 years. At the age of 43 she started suffering systemic hypertension with systolic value around 190–200 mmHg and systolic pressure gradient 70 mmHg. At that time she had a left thoracic trauma with costal fracture. Clinical examination and X-ray showed fibrous impairment of left hemithorax due to previous operation and trauma. Transesophageal echocardiography showed a left ventricular hypertrophy with atrial dilation and detected a recurrent aortic coarctation. CT scan was not advised due to referred contrast medium allergy. MR detected a recurrent coarctation of descending thoracic aorta due to the small caliber of the graft and to intimal hyperplasia at the anastomotic sites (Figure 1). Through a median sternotomy an extra-anatomical bypass from ascending to descending aorta was performed. A 20 mm Dacron graft was employed. By cardiopulmonary bypass and cardioplegic arrest the apex of the heart was elevated to expose the descending thoracic aorta through a longitudinal incision of the dorsal pericardial sac. A sufficient operative field was obtained by caudal retraction of the diaphragm. The distal anastomosis was performed on descending thoracic aorta below the previous bypass, avoiding any esophageal injury; then the proximal anastomosis was placed in the lateral side of ascending aorta. No intra- or postoperative complications were observed. The postoperative pressure gradient was 10 mmHg and the systolic pressure ranged from 130 to 140 mmHg. Post operative MR, six months later, showed a good patency of the graft (Figure 2). 

## 3. Discussion

The recurrent aortic coarctation is often related to the surgical reconstruction performed in the presence of aortic arch hypoplasia. Age, graft material employed, and type of operation condition the recurrence of coarctation. This complication is observed in approximately 10% of patients previously operated. A high incidence is observed in cases of end-to-end anastomosis or subclavian flap repair especially in the presence of arch hypoplasia. The most incidence of recurrent coarctation, about 30%, is detected in patients with prosthetic tubes operated in childhood or adolescence [2], as in fact we observed in our patient. The diagnosis is suspected when the pressure gradient between upper and lower limbs is about 30 mmHg. Careful preoperative investigations including angiography, magnetic resonance (MR) or computed tomography (CT) image scanning, and Echocardiography are useful in deciding the best surgical approach. Transesophageal echocardiography always allows the diagnosis and detects associated heart complications. CT or MRI is able to show clearly the coarctation and to plan the surgical treatment. 

The indication for reoperation is obviously related to the appearance of uncontrolled high blood pressure and left ventricular hypertrophy or dilation. Systemic signs of hypertension or the detection of an aneurysm at the original repair site may suggest the need for reoperation too. In our patient, the diagnosis was confirmed by MRI and the decision of intervention was dictated by the presence of fibrosis at the site of the previous operation and around the Dacron graft inserted. The good general clinical conditions, the age, and the type of previous operation suggested an open repair. As reported by Brown et al. [4], surgical repair of recurrent coarctation produces good and lasting results in the majority of the patients and remains the gold standard. Endovascular treatment is an adequate option in some cases, being less invasive than an open procedure and also with a lower initial cost and a shorter hospital stay. 

Angioplasty and stent placement reduce the rate of aneurysms formation but aortic rupture is a potential lethal complication and often requires an urgent operation: aortic endograft in these circumstances is an effective treatment but a residual stenosis at the site of recurrent coarctation makes it very difficult. However in patients with previous small size tube graft, an open surgical correction is mandatory. The majority of the authors [5] suggest an anatomical aortic reconstruction. The surgical approach is dictated by the site of aortic stenosis and the presence of an arch hypoplasia. If the recurrent obstruction is proximal to the coarctation repair and if the transverse aortic arch remains hypoplastic, a median sternotomy is preferable. If the recurrent coarctation is just proximal or distal to subclavian artery the lesion may be best approached through a redo left thoracotomy. 

In some cases an extra-anatomical bypass from ascending to descending aorta is advised by a median sternotomy. A direct approach to the recurrent coarctation is, in many cases, dangerous due to diffuse fibrous adhesions. In our patient, also, a previous costal fracture at the left hemithorax worsened the local anatomical conditions. In these cases proximal aortic control may be difficult and dissection of fibrous periaortic tissue can produce lung parenchymal lesions. The presence of a thin aortic wall is another possible problem that increases technical difficulties. The extra-anatomical bypass avoids all these problems and, through a median sternotomy, also allows the repair of associated heart pathology. The approach to descending thoracic aorta through the posterior pericardium is easy and the elevation of heart apex allows a correct placement of the graft and a simple anastomosis. In addition, circulatory arrest and circulatory support established between the left atrium and inferior pulmonary vein and the descending aorta is short.

## 4. Conclusion

The extra-anatomical bypass from ascending to descending aorta, for recurrent aortic coarctation, is advised through a median sternotomy and seems to be an effective and safe procedure. It may be considered an effective alternative to the anatomical aortic reconstruction in those cases with recurrent aortic coarctation unfit for endovascular treatment.
